# Early detection of skeletal muscle bioenergetic deficit by magnetic resonance spectroscopy in cigarette smoke-exposed mice

**DOI:** 10.1371/journal.pone.0234606

**Published:** 2020-06-22

**Authors:** Sandra Pérez-Rial, Esther Barreiro, María Jesús Fernández-Aceñero, María Encarnación Fernández-Valle, Nicolás González-Mangado, Germán Peces-Barba

**Affiliations:** 1 Respiratory Research Unit, Biomedical Research Institute—Fundación Jiménez Díaz, Madrid, Spain; 2 Consorcio Centro de Investigación Biomédica en Red de Enfermedades Respiratorias, M.P (CIBERES), Instituto de Salud Carlos III, Madrid, Spain; 3 Respiratory Medicine Department—Muscle Wasting and Cachexia in Chronic Respiratory Diseases and Lung Cancer Research Group, Institute of Medical Research of Hospital del Mar, Barcelona Biomedical Research Park, Barcelona, Spain; 4 Department of Pathology, Hospital Clínico Universitario San Carlos, Madrid, Spain; 5 Nuclear Magnetic Resonance Unit, Bioimaging Research Support Center- Universidad Complutense Madrid, Madrid, Spain; University of Arizona, UNITED STATES

## Abstract

Skeletal muscle dysfunction is a common complication and an important prognostic factor in patients with chronic obstructive pulmonary disease (COPD). It is associated with intrinsic muscular abnormalities of the lower extremities, but it is not known whether there is an easy way to predict its presence. Using a mouse model of chronic cigarette smoke exposure, we tested the hypothesis that magnetic resonance spectroscopy allows us to detect muscle bioenergetic deficit in early stages of lung disease. We employed this technique to evaluate the synthesis rate of adenosine triphosphate (ATP) and characterize concomitant mitochondrial dynamics patterns in the gastrocnemius muscle of emphysematous mice. The fibers type composition and citrate synthase (CtS) and cytochrome c oxidase subunit IV (COX4) enzymatic activities were evaluated. We found that the rate of ATP synthesis was reduced in the distal skeletal muscle of mice exposed to cigarette smoke. Emphysematous mice showed a significant reduction in body weight gain, in the cross-sectional area of the total fiber and in the COX4 to CtS activity ratio, due to a significant increase in CtS activity of the gastrocnemius muscle. Taken together, these data support the hypothesis that in the early stage of lung disease, we can detect a decrease in ATP synthesis in skeletal muscle, partly caused by high oxidative mitochondrial enzyme activity. These findings may be relevant to predict the presence of skeletal bioenergetic deficit in the early stage of lung disease besides placing the mitochondria as a potential therapeutic target for the treatment of COPD comorbidities.

## Introduction

Chronic obstructive pulmonary disease (COPD) is currently considered one of the leading causes of death worldwide and chronic cigarette smoke exposure (CSE) is the main etiological agent. In COPD, systemic manifestations and comorbidities are characteristic features that clearly have a negative effect on the capacity to perform physical exercise and on the quality of life of these patients [1]. Impairment of both has been attributed to muscle dysfunction [2,3]. Loss of muscle mass or atrophy, especially in the lower extremities, is usually associated with impaired function of these muscles in COPD patients. In fact, it was shown that quadriceps muscle dysfunction occurs in a third of patients with COPD, even in the early stages of their disease [4]. In addition, it is known that muscular weakness of the extremities and decreased in muscle mass are important predictors of mortality in COPD, regardless of the severity of the respiratory condition [5,6,7].

The release of inflammatory agents is capable of inducing mitochondrial decoupling and a failure in the expression of the key components of the electron transport chain, which causes severe mitochondrial dysfunction and a significant decrease in mitochondrial ATP production. A key feature of skeletal muscle dysfunction is a disrupted capacity for oxidative metabolism, which is believed to contribute to patient fatigue, decreased metabolic function, and muscle mass loss [8,9].This dependence on oxidative metabolism underlines the critical role of muscle mitochondria in metabolic homeostasis. The metabolic capacity of skeletal muscle is an important determinant in lung disease. Skeletal muscle needs large rates of cellular metabolism and energy production. Mitochondria are essential to maintain the homeostasis of skeletal muscle energy. Oxidative phosphorylation is a metabolic process that uses the energy released by the oxidation of nutrients to produce adenosine triphosphate (ATP) [10,11]. Non invasive 31-phosphorus magnetic resonance spectroscopy (^31^P-MRS) allows measurements of physiological biomarkers in intact systems and has shown its ability to detect mitochondrial dysfunction in a murine model of cancer cachexia [2,3]. With this methodology, researchers can measure the net ATP synthesis rate of skeletal muscle catalyzed by mitochondrial ATPase [12]. However, it has never been used to detect early muscle dysfunction in COPD animal models.

Skeletal muscle consists of slow and fast fibers that differ in the composition of the contractile proteins, the oxidative capacity and the substrate used for the ATP production. Myosin is an important contractile protein, which converts chemical energy into mechanical energy by ATP hydrolysis. Classically, the types of fibers are classified by the expression of the isoform of the heavy chain of myosin in type I (slow-contraction) and type II (fast-contraction), which can differ substantially in the mitochondrial content and in the oxidative capacities of metabolic enzymes. In humans, the femoral quadriceps (vastus lateralis) is a predominantly fast-fiber muscle consisting of approximately 40% type I fibers and 60% type II fibers, while the anterior tibialis is a predominantly slow-moving muscle which consists of approximately 70% of type I and 30% of type II fibers [13,14]. Accordingly, in rats following ischaemia–reperfusion of its lower limb, it was observed that mitochondrial dysfunction increased more in the gastrocnemius muscle (mainly glycolytic), than in the soleus muscle (mainly oxidative) [15]. Interestingly, a stronger association was observed between physical activity and oxidative capacity in the vastus lateralis compared to the tibialis anterior, suggesting that the former is more sensitive to inactivity and needs more stimuli, which explains why it is more fatigable [16].

Mitochondrial respiration is the most efficient bioenergetic mechanism in skeletal muscle and is linked to oxidative phosphorylation, which ultimately allows mitochondria to generate ATP. Abnormalities in muscle mitochondrial content, morphology and function have been reported in various muscle wasting conditions, including those in limb muscles in COPD patients [17]. In addition, in these patients the activities of key enzymes in oxidative metabolism was evaluated, such as citrate synthase (CtS) and cytochrome c oxidase subunit IV (COX4) were evaluated [18]. The CtS activity was used as a marker of mitochondrial content or density, since it catalyze the conversion of acetyl-coenzyme and oxaloacetate into citrate and coenzyme A and serves as a marker for intact mitochondria [19,20,21,22]. The COX4 activity was evaluated by estimating the oxidation rate of ferrocytochrome c to ferricytochrome c. In the skeletal muscle of COPD patients, several studies have shown a decrease in the IV complex [23].

We hypothesized that chronic CSE promotes mitochondrial uncoupling and causes a reduction in ATP synthesis in the lower limb muscles. The first objective of this study was to evaluate the rate of ATP synthesis in a mouse emphysema model by using ^31^P-MRS *in vivo*. Our second objective was to characterize the concomitant mitochondrial dynamic patterns in the skeletal muscle of emphysema *versus* intact control mice.

## Material and methods

### Experimental design and ethics

A total of 20 male, 10-week-old C57BL/6J mice (Charles River Laboratories, France) were housed in the Core Facility at the Biomedical Research Institute—Fundación Jiménez Díaz (IIS-FJD), Madrid. The protocols were approved by the Animal Welfare and Ethic Committee from IIS-FJD (PROEX 322/14), in agreement to the Spanish Royal Decree 53/2013 based on the European Union normative (2010/63/UE) concerning animal protection for research purposes. All surgery was performed under anesthesia and all efforts were made to minimize animal suffering. The body weights of all animals of each group were determined immediately before and after the 7 months of study. The percentage of body weight gain was calculated as: [(body weight at 7 months − body weight on day 0)/body weight on day 0] × 100.

### Chronic cigarette smoke exposure—induced emphysema model

Mice were divided into controls, air-exposed (CTL, n = 10) and smokers, cigarette smoke-exposed (CSE, n = 10). Smoking animals were exposed to a mainstream CSE of 4 unfiltered cigarettes (3R4F, University of Kentucky, Lexington, KY) per day (15 minutes per cigarette with 5 minutes smoke free intervals between them), 2 cigarettes in the morning and 2 cigarettes in the afternoon, 5 days a week during 7 months. Mainstream CSE was generated by an exposure system of own manufacture and was drawn into the chamber using a peristaltic pump following the previously published methodology [24] that reaching concentrations of 250 mg TPM/m^3^ (Dust Track Model 8520, TSI Inc.). Non-smoking mice were exposed to room air. Effectiveness of cigarette smoke exposure was assessed spectrophotometrically by measuring blood levels of carboxyhemoglobin (COHb) in animals randomized following previously published methodology [24]. The mean percent COHb of control mice was 0.3 ± 0.06% and of mice exposed to smoke chamber was within nontoxic 19.2 ± 0.2% value, confirming the correct exposure to tobacco smoke.

### Sacrifice and sample collection

After seven months of exposure, mice from both experimental groups were sacrificed by intraperitoneal injection of sodium pentobarbitone, 100 mg/kg body wt (Abbot Laboratories, Lake Forest, IL, USA). The lung tissue was removed from the cardiopulmonary block and the lungs were fixed by filling them with 4% buffered formalin solution at a constant airway pressure of 25 cmH_2_O for 24 h. The fixed lungs were kept in the formalin solution until they were embedded in paraffin and sections 5-μm-thick were cut using a rotary microtome HM325 (Microm International GmbH). The gastrocnemius muscles were collected from the hind limbs using standardized dissection methods and cut into 5 μm of the samples embedded in paraffin and fixed with formalin.

### Morphometric analyses of the lungs

The fixed lung slides were stained with hematoxylin-eosin (Sigma Aldrich Química S.L.U.) according to standard protocols and examined under a light microscope (Olympus BX40) with digital recorder (Leica DFC290, Leica Microsystems). Areas with large blood vessels or airways >50 μm, or with obvious artifacts were excluded. The degree of emphysema was evaluated by measuring in the alveolus the mean linear intercept (L_M_, μm) and the alveolar internal area (AIA, μm^2^) following previously published methodology [24].

### Lung function of living mice

The animals were initially anesthetized with the isoflurane inhalation anesthetic (FORANE, AbbVie Farmacéutica, S.L.U.) and subsequently with a ketamine/xylacine combination (1/3), 0.1 ml/10 g body wt i.p. (KETOLAR, Pfizer and ROMPUN, Bayer). Mice were tracheotomized and connected to the ventilator according to the previously published methodology [24]. Changes in quasistatic lung compliance (C_L_, ml/cmH_2_O) and maximum expiratory volume (V_max_, ml) at a pressure of 25 mbar after pressure-controlled ventilation were studied for each of the experimental groups.

### *In vivo* mouse skeletal muscle ^31^P-MRS

The mitochondrial muscle ATP synthesis rate was evaluated *in vivo* in mice by ^31^P-MRS using a Biospec BMT 47/40 spectrometer (Bruker, Ettlingen, Germany) operating at 4.7 Tesla, equipped with a 6-cm gradient system and a home-made double-tuned (^1^H and ^31^P) 1.6-cm surface coil. In brief, the animal was anesthetized with a mixture of isoflurane (FORANE, AbbVie Farmacéutica, S.L.U.) and oxygen (3% oxygen at 1.5 L/min for induction and 1.0–1.5% at 1.0 L/min during experiments), both hind limbs were shaved and the mouse was positioned prone in a polymethacrylate cradle that exposed the muscles to the surface of the coil to ensure a consistent and reproducible positioning for each study. Coil geometries were optimized to match the size of the mouse and to ensure the proximity of the muscle to the coil for a high signal-to-noise ratio, while avoiding the occlusion of blood flow at the extremity of the mouse. Respiration was monitored throughout the experiment and body temperature was maintained at 37°C, using a 1025 MR-compatible small rodent monitoring and gating system (SA Instruments, Inc., New York, USA). This experimental configuration eliminates the movement of the region of interest and ensures the acquisition of high signal/noise spectra. First, the adjustment of the homogeneity and the fast images, to ensure the localization, were performed with the surface coil tuned in ^1^H, then the tune was changed to ^31^P and the spectroscopic experiments were carried out. Phosphorus spectroscopy was performed on each animal with a repetition time of 2500 ms, a flip angle of 90° and 1536 scans. By using Nuts software (AcornNMR, Livermore, CA, USA) for spectra processing, we are able to estimate muscle Pi content. Chemical shifts were expressed relative to phosphocreatine (PCr) resonance, to which zero shift was assigned. The pH was estimated by the chemical shift of the Pi signal relative to the PCr signal. Signals in ^31^P NMR spectra were determined quantitatively by fitting the peak in question (after phase and baseline corrections) to a Lorentzian function, using the LCModel method [25,26]. Differences in chemical shift between the γ, α and β-phosphorus nuclei of ATP were used to assess the amount of Mg^2+^ ions bound to ATP and to estimate their levels. Peak area of each signal was expressed as a percentage of the total ^31^P-MRS signal (the sum of all the resonances from 25 ppm to -25 ppm). This procedure eliminates the dependence of these measurements on experimental parameters such as the mouse size, local magnetic field homogeneity, etc. These experiments were carried out at the Research Center of Nuclear Magnetic Resonance and Electronic Spin of the Multidisciplinary Institute of the Complutense University of Madrid.

### Analysis of muscle fiber type

For immunohistochemical procedures, the slides of the gastrocnemius muscle were incubated with mouse monoclonal antibodies against myosin heavy chain type I (MyHC-I, slow fiber, clone NOQ7.5.4D) and type II (MyHC-II, fast fiber, clone MY-32), Sigma Aldrich Química S.L.U. An equal number of slides underwent the same procedures without the primary antibodies (negative controls). The analysis was carried out using the peroxidase-conjugated avidin/biotin complex method. The gastrocnemius slides were deparaffinized by incubation at 65°C for 30 min and rehydrated in a graded xylene-alcohol series. Antigen retrieval was performed using a solution of 1 mM EDTA in citrate buffer (pH 6.0) at high temperature (95°C) in PT-Link (Dako, Denmark). Endogenous peroxidase was inhibited using 3% hydrogen peroxide in methanol. The nonspecific antigen-antibody reaction was blocked through the incubation of the sections in a serum-free solution (Dako, Denmark) for 30 min. Gastrocnemius sections were incubated for 30 min with a 1:400 dilution of myosin antibody. Subsequently, all sections were treated with anti-Ig horseradish peroxidase-conjugated polymer (EnVision, Dako, Denmark) to detect antigen-antibody reaction, which was visualized as a brown precipitate following the application of 3, 3-diaminobenzidine for 10 min and contrasted with hematoxylin for 5 min. The slides were then rehydrated and mounted for observation under light microscopy. The fiber types (I and II) were individually counted by pathologists and expressed as a percentage (number of positive stained cells over the total number of cells). The cross-sectional area of total fibers type was determined and expressed as total area (μm2).

### Muscle metabolic profile

Gastrocnemius muscles were collected from all mice and snap frozen for later stored at −80°C until further use. Muscle tissue homogenization and mitochondrial isolations were always performed at 4°C according to the manufactured instructions. Samples were homogenized using a tapered tissue grinder (Wheaton™). In general, 50 mg of whole muscle tissue was incubated for 3 minutes and placed into the homogenizer with 10 volumes of hard tissues extraction buffer containing 0.25 mg/ml trypsin and then spin down for a few seconds. Samples were then transferred into a new tube with 8 volumes of the extraction adding 10 mg/ml bovine serum albumin and centrifuged at 10,000 g for 10 min. The supernatants were collected and placed into new tubes, while the pellets were resuspended with the storage buffer. The homogenization procedure was repeated again with the resuspended pellets. The supernatant obtained from the second centrifugation was added to that of the first, thus giving the final supernatant of the sample. The resulting mitochondrial extracts were assayed using a specific colorimetric assays kit for citrate synthase (CtS) and cytochrome oxidase c subunit IV (COX4) activity (Sigma Aldrich Química S.L.U.). CtS react with the substrate mix to form an intermediate, which subsequently reacts with the developer to generate the colored product monitored at 412 nm. The rate of color development is proportional to the enzymatic activity. Oxidation of cytochrome c was monitored at 550 nm. The specific activity of the mitochondrial enzyme was expressed as nmol/min/μl. The COX4 activity is also expressed as a ratio to CtS to compensate for mitochondrial enrichment in the cell samples.

### Data analysis

The data are represented as mean ± standard deviation (SD). Values of p <0.05 are considered statistically significant. Variations in body weight over time are explored using Student´s t-test. The nonparametric U-Mann-Whitney test is used for comparisons between groups of all the biological variables studied, followed by Monte Carlo’s exact method, using the software from Statistical Package for the Social Science (IBM SPSS).

## Results

### Body weight gain reduced after CSE

**Fig 1:**
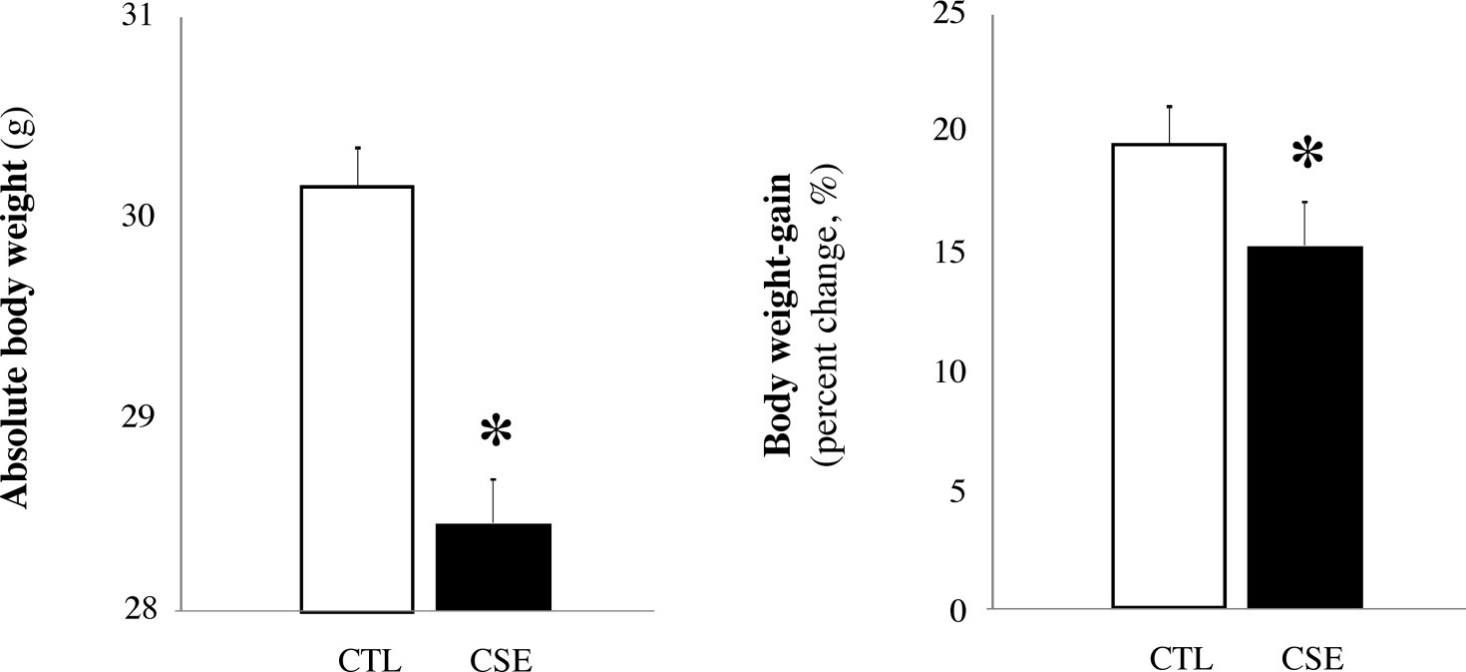
Body weight gain after CSE. Bars represents absolute body weight (g) and body weight-gain (percent change, %) of air-exposed group (CTL, white bar) and cigarette smoke-exposed mice (CSE, black bar). Data are represented as mean ± SD, *p≤0.05.

Body weight gain of CSE mice and CTL mice were assessed at 7 months of the study (Fig 1). We observed that body weight gain in CSE mice was significantly reduced compared to air-exposed mice: 19.9 ± 0.5% in CTL mice (30.2 ± 0.3 g) *versus* 15.4 ± 0.3% in CSE mice (28.5 ± 0.4 g), p = 0.029.

### Morphometric studies in lung tissue confirm emphysema

**Fig 2:**
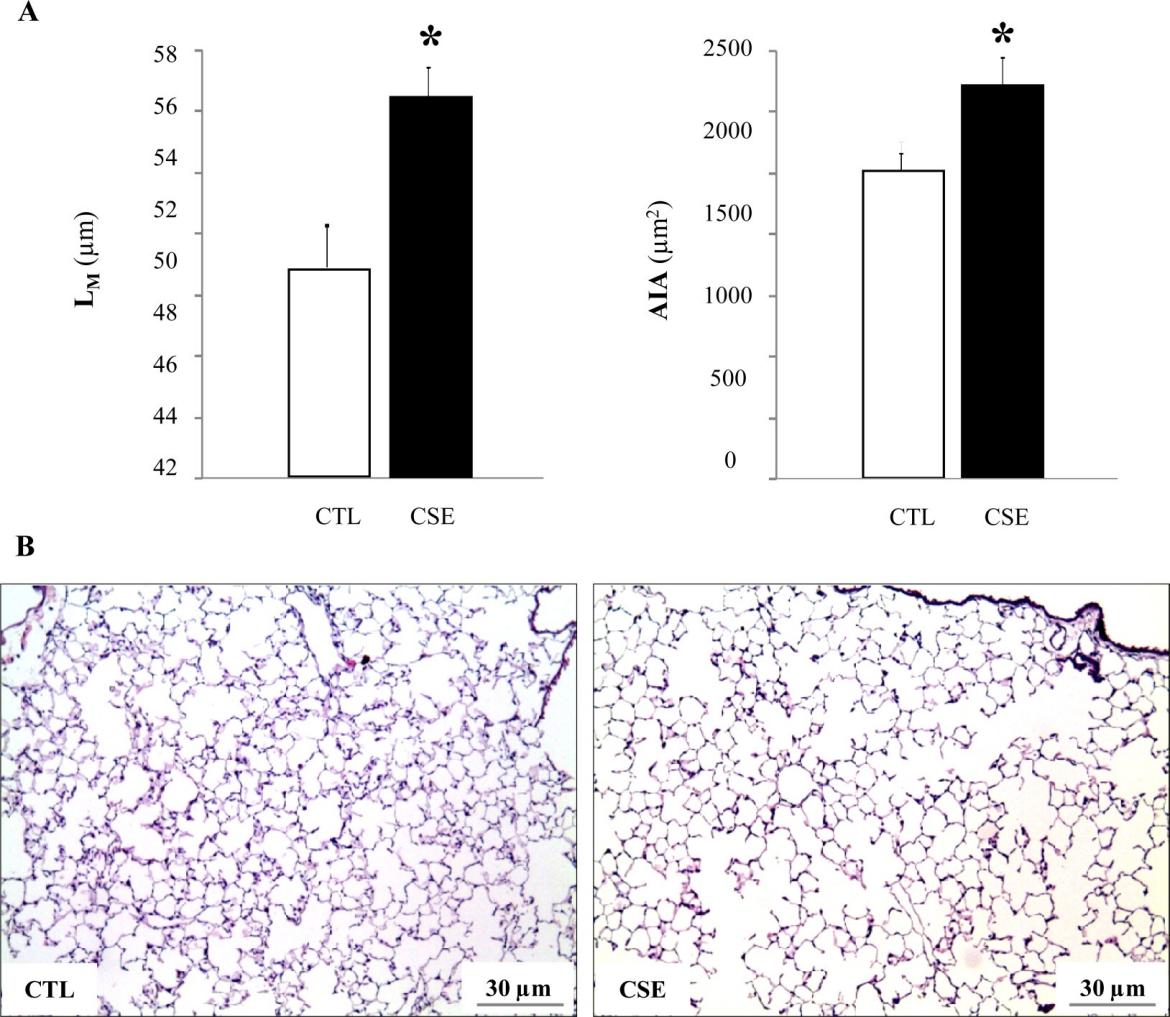
Alveolar space in emphysematous mice. (A) Mean values of the mean linear intercept (L_M_, μm) and alveolar internal area (AIA, μm^2^) in the lungs of the cigarette smoke-exposed mice (CSE, black bar) compared to air-exposed group (CTL, white bar). Note that the alveolar space is enlarged in the emphysematous mice. Data are expressed as mean ± SD, *p≤005. (B) Representative images (x10 magnification) corresponding to lung samples (30 μm calibration bar).

To evaluate the degree of lung damage and the development of emphysema caused by CSE, the mean linear intercept (L_M_, μm) and alveolar internal area (AIA, μm^2^) were measured. The CTL mice had normal alveolar architecture in contrast to the CSE mice that presented an enlarged alveolar space (L_M_ = 50.1±0.55 μm *versus* 56.8 ± 0.62 μm, respectively, p = 0.010 and AIA = 1853.2 ± 20.6 μm^2^ *versus* 2372.8 ± 31.4 μm^2^, respectively, p = 0.019) (Fig 2A). Representative histological sections of lungs in CTL and CSE mice are shown in Fig 2B.

### Impairment of lung function in emphysematous mice

**Fig 3:**
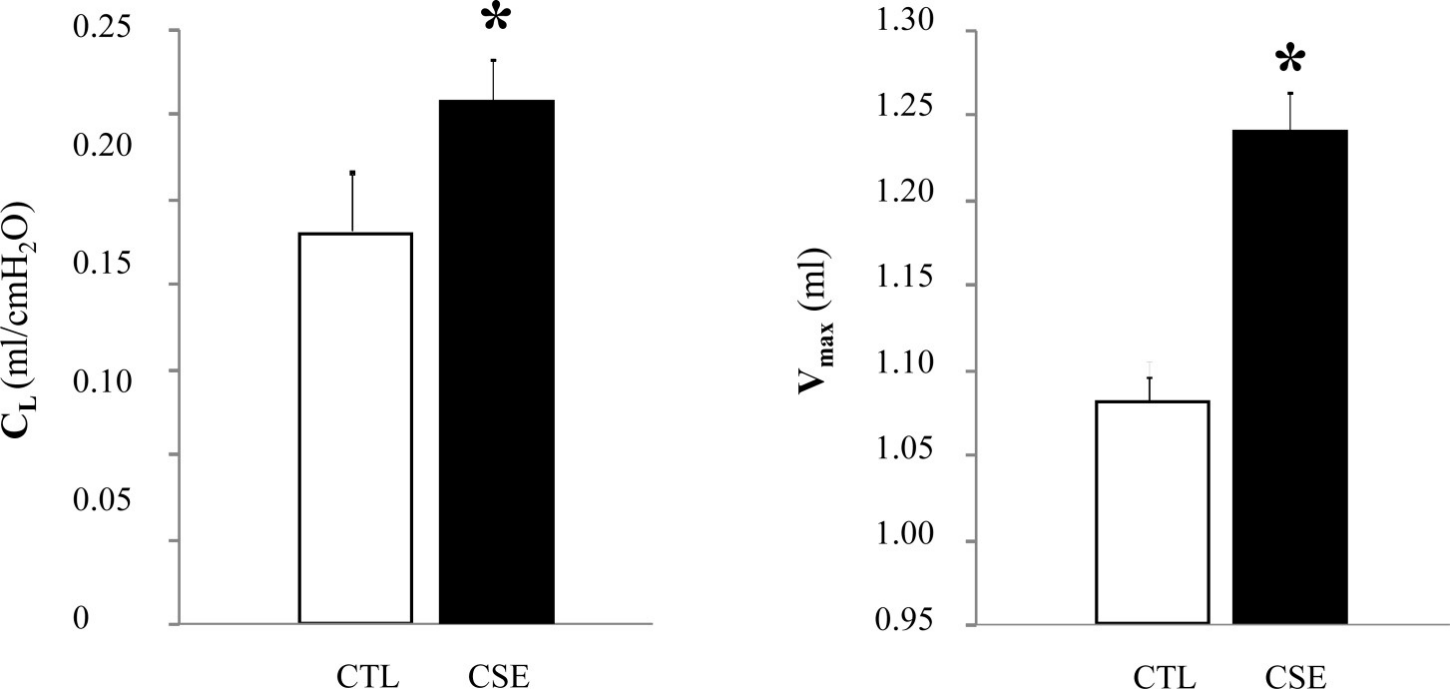
Lung function of emphysematous mice. Changes in lung compliance (C_L_, ml/cmH_2_O) and maximum expiratory volume (V_max_, ml) in the lungs of cigarette smoke-exposed mice (CSE, black bar) compared to air-exposed group (CTL, white bar). Data are expressed as mean ± SD, *p≤005.

Pulmonary emphysema induced by CSE led to a significant increase in C_L_ and V_max_ at 25 mbar (Fig 3) of CSE group (0.22 ± 0.005 ml/cmH_2_O (p = 0.010) and 1.24 ± 0.03 ml (p = 0.004), respectively) compared to CTL group (0.16 ± 0.003 ml/cmH_2_O and 1.08 ± 0.02 ml).

### Reduction of ATP synthesis rate in skeletal muscle

**Fig 4:**
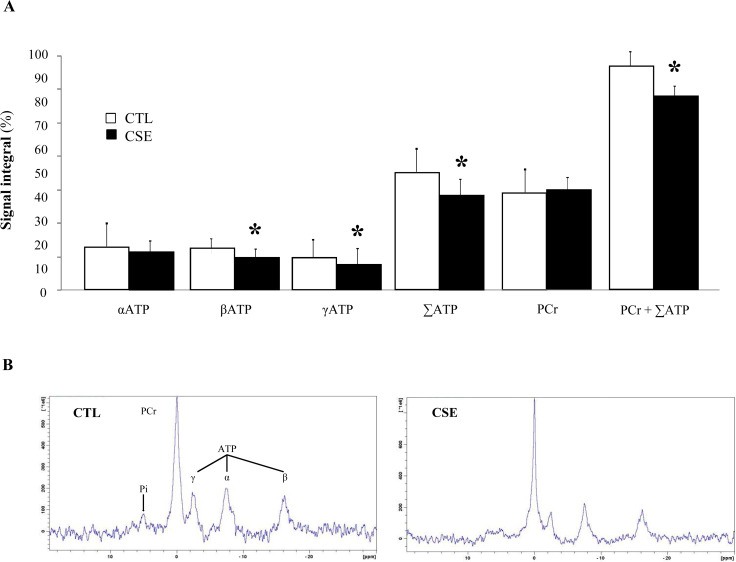
ATP synthesis rate in skeletal muscle of emphysematous mice. (A) *In vivo* ^31^P-NMR spectra performed on the hind limb skeletal muscle of conscious mice. The difference in spectra between air-exposed group (CTL, white bar) and cigarette smoke-exposed mice (CSE, black bar) is expressed as percentage of CTL muscle, set at 100%. Data are expressed as mean ± SD, *p≤005. (B) Representative ^31^P-NMR spectra acquired in CTL (left) and CSE (right) mice.

Muscle energy status was evaluated by ^31^P-MRS. Peaks of high-energy phosphate metabolites (PCr, ATP) and Pi were respectively identified in spectra measured from cigarette exposed and non exposed hind limb muscle. Fig 4 shows ^31^P-NMR spectra representative of CTL and CSE mice. It was measured by values of the integral of each signal in percentage with respect to the integral of the total spectrum. Compared to CTL mice, pulmonary emphysema induced by chronic CSE resulted in a significant decrease in βATP (18.53 ± 1.24% *versus* 13.86 ± 0.87%, p = 0.017), γATP (13.82 ± 1.02% *versus* 11.58 ± 0.8%, p = 0.041), ∑ATP (49.75 ± 1.03% *versus* 40.62 ± 1.24%, p = 0.002) and PCr + ∑ATP (94.39 ± 0.69% *versus* 83.03 ± 0.95%, p = 0.035). No changes were observed in the integral intensity of the PCr and αATP peaks (in terms of signal-to-noise ratio) in CSE hind limbs.

### Skeletal muscle structure: Composition in fiber type

**Fig 5:**
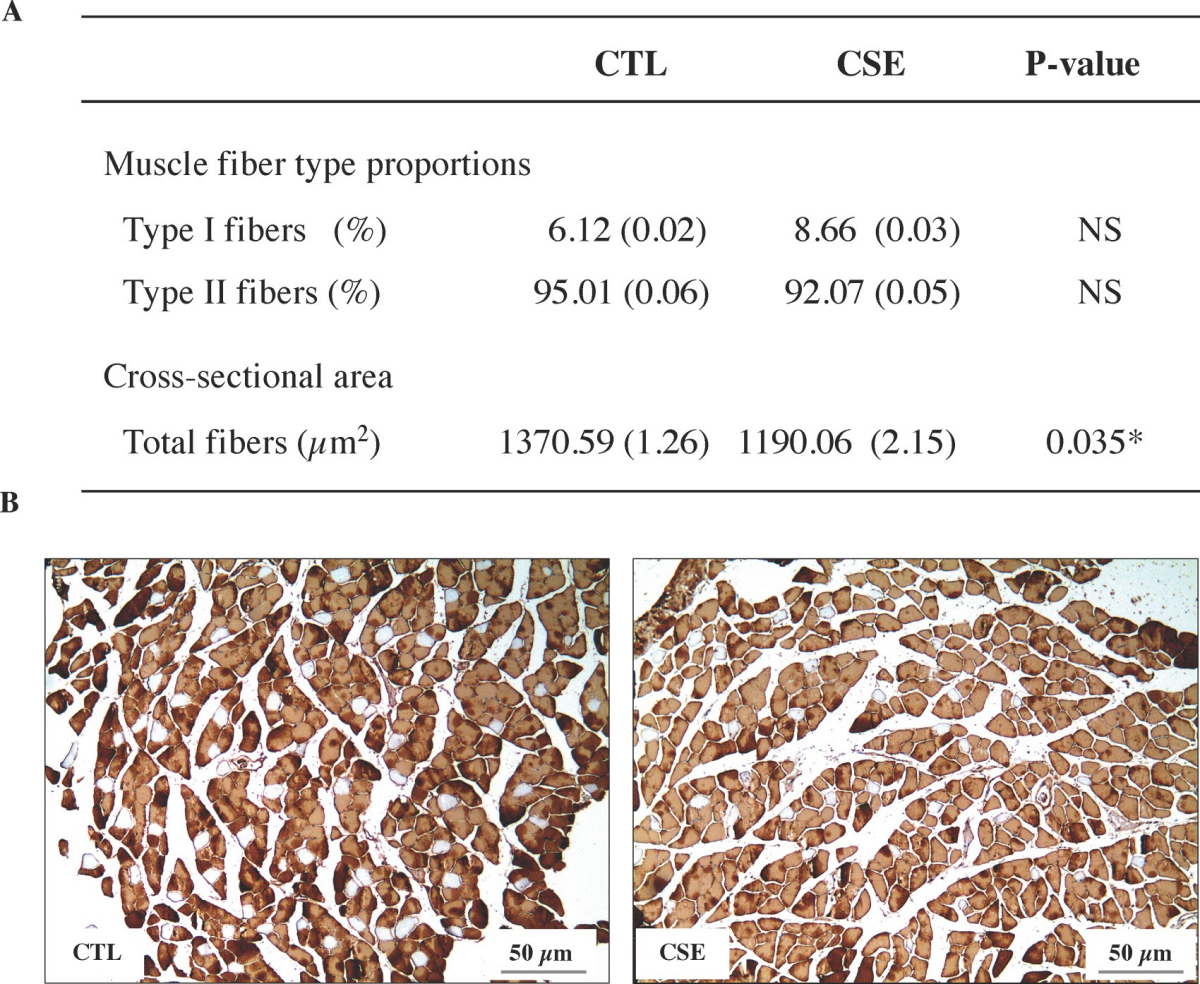
Gastrocnemius muscles structure of emphysematous mice. (A) Percentages of fibers type I and II and total fibers in the cross-sectional area of the gastrocnemius of air-exposed (CTL) and cigarette smoke-exposed (CSE) mice. Data are represented as mean ± SD, NS, not significant, *p≤0.05. (B) Immunohistochemistry representative images (x10 magnification) of myosin stained fibers in gastrocnemius muscles of CTL (left) and CSE (right) group. Myofibers positively stained with the anti-MyHC type II antibody appear brown (calibration bar 50 μm). Type I fibers appear unstained.

The content of MyHC in hind limb muscles after chronic air or cigarette smoke exposure is given in Fig 5. There was a non-statistically significant increase in the proportion of type I fibers in the gastrocnemius muscle after exposure to cigarette smoke with respect to the CTL group. While in type II there was a small decrease not statistically significant (Fig 5A). However, there was a significant decrease in the number of fiber in the total cross-sectional area in CSE group compared to CTL group (1190.06 ± 2.15 μm2 *versus* 1370.59 ± 1.26 μm2, respectively, p = 0.035). Histological characteristics of a representative gastrocnemius muscle section are shown. Immunohistochemical staining (DAB, brown) are given for myosin heavy chain type I and II (brown label) in air-exposed (CTL) and cigarette smoke-exposed (CSE) mice (Fig 5B).

### Mitochondrial enzymes activity impairment

**Fig 6:**
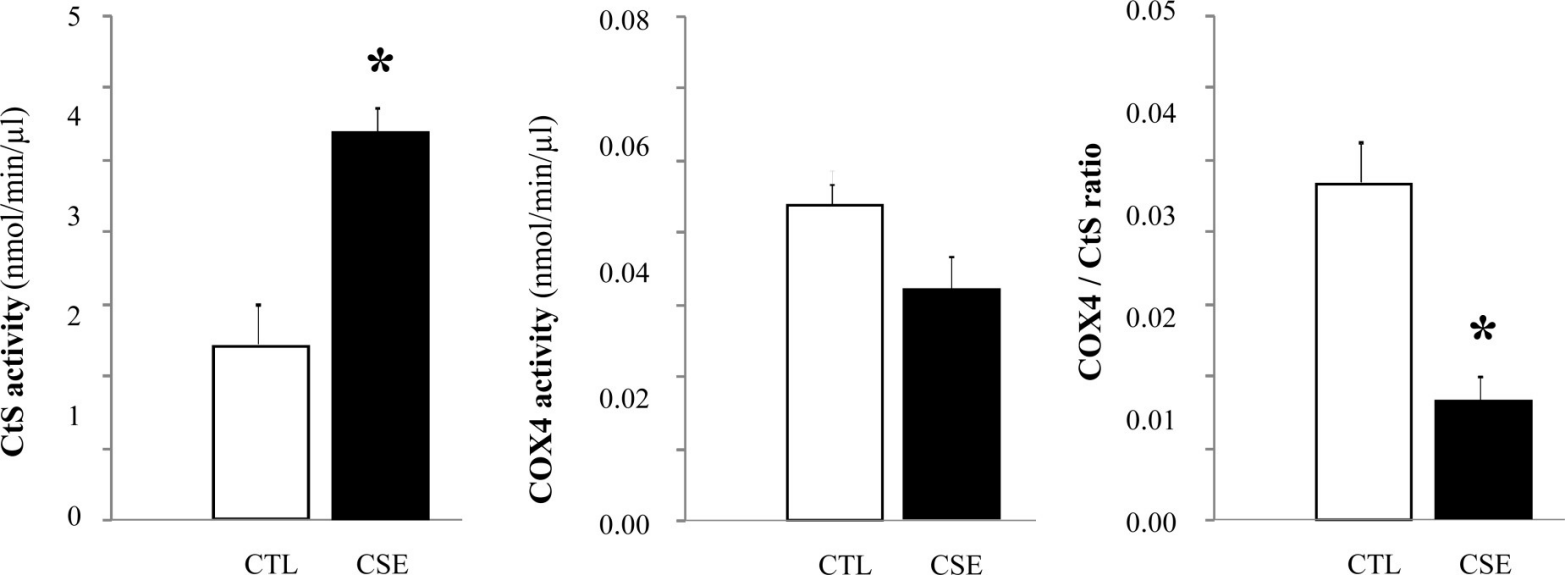
Mitochondrial enzymes activity in the skeletal muscle of emphysematous mice. Citrate synthase (CtS) and cytochrome c oxidase subunit IV (COX4) activity in mitochondria isolated from gastrocnemius of air-exposed group (CTL, white bar) and cigarette smoke-exposed mice (CSE, black bar) was evaluated by spectrophotometric methods. Data are represented as mean ± SD, *p≤0.05.

The results of the muscular metabolic profile of oxidative enzymes are shown in Fig 6. Compared with CTL mice, citrate synthase (CtS) activity was significantly increased in gastrocnemius muscle of CSE mice (1.76 ± 0.02 nmol/min/μl *versus* 3.93 ± 0.03 nmol/min/μl, respectively; p = 0.016). Cytochrome c oxidase subunit IV (COX4) activity decreased, although not significantly. The activity relationship COX4 to CtS decreased significantly in CSE mice compared to CTL mice (0.012 ± 0.0001 nmol/min/μl *versus* 0.033 ± 0.0002 nmol/min/μl, respectively; p = 0.014), mainly due to this increase in activity of the CtS.

## Discussion

The main observation of this study is that, in this murine early COPD model, chronic CSE results not only in mild emphysema and impaired lung function, but also in gastrocnemius skeletal muscle dysfunction in emphysematous mice, which is directly related to mitochondrial enzyme dysfunction in the limb muscles of these mice. These findings would explain the reduced rate of muscle ATP synthesis and the decreased in the COX4/CtS activity ratio by the increase in oxidative capacity (CtS activity) in the gastrocnemius of mice exposed to chronic CSE compared to air-exposed control mice. Taken together, these data suggest that mitochondrial uncoupling occurs early in the development of COPD and, therefore, draws to mitochondria as a possible pharmaceutical target for treatment of this disease.

One of the most outstanding characteristics of the complaints of patients with COPD is the reduction of exercise tolerance, caused in part by the structural and functional deterioration of the skeletal muscles [27]. Previous results published by our group show how chronic exposure to cigarette smoke induces increased oxidative stress levels in their gastrocnemius, greater oxidative modifications on muscle proteins and systemic inflammation [26]. Proteins involved in ATP production were carbonylated in limb muscles and blood tumor necrosis factor (TNF)-alpha levels were significantly greater in cigarette smoke mice than in control animals [28]. Altered metabolism of high-energy phosphate in muscles seems to contribute to this exercise intolerance [29]. However, literature is not consistent with respect to the distribution of muscle atrophy in smokers. Skeletal muscle fibers can change from one fiber type to another, depending on the demand for physical activity. That change in fiber type is observed in the diaphragm of COPD patients, where a fast-to-slow fiber switch phenotype is associated with a significantly reduced diaphragm force [30]. However, a slow-to-fast fiber type shift is a constant finding in lower extremities muscles of patients with COPD, in which fiber-type transitions seem to play an important role [31]. In contrast, there are no changes in the bicep brachii of COPD patients compared to controls of the same age [32,33]. Inconsistency also appears in the few animal studies that have been reported. Several studies showed that in mice with emphysema, more prominent signs of muscle mass wasting and loss of MyHC were observed in the diaphragm than in the limb muscle [34]. However, other studies in line with our results do not find significant differences in the proportion of muscle fiber type I and II in the gastrocnemius of emphysematous mice [35,36].

Weakness and muscle wasting predict morbidity and mortality in patients with COPD [37]. However, the mechanisms that potentially correlate muscle dysfunction with lung disease have not yet been fully clarified. To obtain information on skeletal muscle energy metabolism in response to cigarette smoke exposure, the energy status index (PCr and ATP) was measured using ^31^P-MRS. This imaging technique is currently the only available that allows the non-invasive measurement of the main phosphorylated compounds involved in the metabolism of muscle energy [1]. ATP is a substrate for all cellular reactions that consume energy, since its hydrolysis provides free energy. PCr behaves as a compound of energy storage and serves as an energy carrier molecule in the creatine kinase/PCr energy shuttle [38]. Thus, the PCr/ATP ratio has been used to index the energy status of the muscle. On the other hand, the loss of the peripheral muscle oxidative phenotype in COPD is a constant finding, and it is believed that it is involved in the increase of muscle fatigue in these patients. In the present investigation, it has been clearly demonstrated for the first time, that the early stage of lung disease can be detected *in vivo* by the analysis of skeletal muscle and its decrease in the ATP synthesis rate.

In this study it has been demonstrated that the decrease in the synthesis of muscle ATP is due to a decrease in the COX4/CtS activity ratio in the gastrocnemius of emphysematous mice, as a consequence of an increase in the oxidative activity of CtS. This reduction in the activity rate of the enzymes involved in oxidative metabolism is in line with previous studies in which alterations in mitochondrial function were also detected in the muscles of moderate to severe COPD patients [39]. However, some authors attribute this decrease in the COX4/CtS activity ratio to a decrease in the activity of CtS in gastrocnemius of mice exposed to cigarette smoke [20,22]. This discrepancy has been explained by some authors due to the presence of two different muscle profiles within the same gastrocnemius muscle, with pronounced morphological and functional differences in the subtypes of muscle fibers and in their oxidative capacity [37]. A superficial region consisting predominantly of oxidative muscle fibers; called "white" gastrocnemius and another deep region, with the presence of high oxidative capacity, called "red" gastrocnemius. Thus, our sampled region would correspond with the “red” gastrocnemius, with high oxidative capacity and abundance of type II fibers.

Interestingly, the activity of CtS enzyme was significantly greater than that of the controls if only severe COPD patients were considered [37]. As some authors have already pointed out, this tendency towards the increase of CtS activity observed in the gastrocnemius muscle could be explained as an adaptive response to maintain an appropriate level of daily activity and muscle recruitment, under an increase in respiratory load [40]. This means that the slow-twitch and fatigue-resistant muscle fibers are activated before the fast-twitch muscle fibers and less fatigue resistance. The degree of fatigue is minimized, first using fatigue-resistant muscle fibers and using only fatiguing fibers when large forces are needed.

However, other authors do not find this decrease in CtS activity in the tibialis anterior muscle [41,42], but an increase in this activity in the deltoid muscle [43] in COPD patients. It is likely that the relative inactivity that occurs frequently in patients with severe COPD affects a locomotor muscle more than a non-locomotor muscle. This latter phenomenon could explain the fact that a decrease in oxidative capacity has been found in the femoral quadriceps muscle, but not in the deltoid or tibialis anterior muscle of patients with COPD [44]. Mitochondrial disruption and decreased ATP synthesis rate were also shown in the limb muscles of cancer induced cachectic mice [43]. Our findings clearly point to a role of smoking in the loss of muscular oxidative phenotype and suggest that the reduction in the ATP synthesis is related to mitochondrial dysfunction.

In summary, the early COPD model used in this study is certainly a model which would be equivalent to mild emphysema in patients, with no data of chronic bronchitis, reminiscent of the phenotype of emphysema in COPD that is specifically associated with muscle dysfunction. These systemic effects are clinically relevant in the evolution of the disease in patients. Our results suggest the presence of muscular dysfunction in the early stages of emphysema. Due to its prognostic value, we could suggest the assessment of skeletal muscle dysfunction in the routine evaluation of patients with emphysema, even in the early stages.
